# Voluntary Medical Male Circumcision: Modeling the Impact and Cost of Expanding Male Circumcision for HIV Prevention in Eastern and Southern Africa

**DOI:** 10.1371/journal.pmed.1001132

**Published:** 2011-11-29

**Authors:** Emmanuel Njeuhmeli, Steven Forsythe, Jason Reed, Marjorie Opuni, Lori Bollinger, Nathan Heard, Delivette Castor, John Stover, Timothy Farley, Veena Menon, Catherine Hankins

**Affiliations:** 1 United States Agency for International Development, Washington, District of Columbia, United States of America; 2 Futures Institute, Glastonbury, Connecticut, United States of America; 3 Centers for Disease Control and Prevention, Atlanta, Georgia, United States of America; 4 UNAIDS, Geneva, Switzerland; 5 Office of the U.S. Global AIDS Coordinator, United States Department of State, Washington, District of Columbia, United States of America; 6 World Health Organization, Geneva, Switzerland; 7 Futures Group, Washington, District of Columbia, United States of America; 8 Joint United Nations Programme on HIV/AIDS, Geneva, Switzerland; Centers for Disease Control and Prevention, United States of America

## Abstract

**Background:**

There is strong evidence showing that voluntary medical male circumcision (VMMC) reduces HIV incidence in men. To inform the VMMC policies and goals of 13 priority countries in eastern and southern Africa, we estimate the impact and cost of scaling up adult VMMC using updated, country-specific data.

**Methods and Findings:**

We use the Decision Makers' Program Planning Tool (DMPPT) to model the impact and cost of scaling up adult VMMC in Botswana, Lesotho, Malawi, Mozambique, Namibia, Rwanda, South Africa, Swaziland, Tanzania, Uganda, Zambia, Zimbabwe, and Nyanza Province in Kenya. We use epidemiologic and demographic data from recent household surveys for each country. The cost of VMMC ranges from US$65.85 to US$95.15 per VMMC performed, based on a cost assessment of VMMC services aligned with the World Health Organization's considerations of models for optimizing volume and efficiencies. Results from the DMPPT models suggest that scaling up adult VMMC to reach 80% coverage in the 13 countries by 2015 would entail performing 20.34 million circumcisions between 2011 and 2015 and an additional 8.42 million between 2016 and 2025 (to maintain the 80% coverage). Such a scale-up would result in averting 3.36 million new HIV infections through 2025. In addition, while the model shows that this scale-up would cost a total of US$2 billion between 2011 and 2025, it would result in net savings (due to averted treatment and care costs) amounting to US$16.51 billion.

**Conclusions:**

This study suggests that rapid scale-up of VMMC in eastern and southern Africa is warranted based on the likely impact on the region's HIV epidemics and net savings. Scaling up of safe VMMC in eastern and southern Africa will lead to a substantial reduction in HIV infections in the countries and lower health system costs through averted HIV care costs.

*Please see later in the article for the Editors' Summary.*

## Abstract 2

Emmanuel Njeuhmeli and colleagues estimate the impact and cost of scaling up adult medical male circumcision in 13 priority countries in eastern and southern Africa, finding that reaching 80% coverage and maintaining it until 2025 would avert 3.36 million new HIV infections.

## Editors' Summary

**Background:**

Every year, about 2.5 million people (mainly in sub-Saharan Africa) become infected with HIV, the virus that causes AIDS. There is no cure for HIV/AIDS. Consequently, prevention of HIV transmission is very important. Because the most common HIV transmission route is through unprotected sex with an infected partner, individuals can reduce their risk of HIV infection by abstaining from sex, by having only one or a few partners, and by using male or female condoms. There is also strong evidence that voluntary medical male circumcision (VMMC)—the removal of the foreskin, the loose fold of skin that covers the head of the penis—reduces the heterosexual acquisition of HIV in men by about 60%. In 2007, the World Health Organization (WHO) and the Joint United Nations Programme on HIV/AIDS (UNAIDS) recommended that VMMC should be offered to men as part of comprehensive HIV risk reduction programs in settings with generalized HIV epidemics and low levels of male circumcision. They also prioritized 13 countries in eastern and southern Africa for VMMC program scale-up.

**Why Was This Study Done?:**

The impact of VMMC scale-up in terms of HIV infections and AIDS deaths averted (epidemiologic impact) is expected to be large, and the intervention should also reduce the costs associated with the treatment, care, and support of infected individuals. However, VMMC scale-up will require substantial funding and considerable effort by countries—many of which have weak health systems and limited resources—to train personnel, equip facilities, and provide the necessary commodities. To support planning for VMMC scale-up, the United States Agency for International Development Health Policy Initiative has collaborated with UNAIDS to develop the Decision Makers' Program Planning Tool (DMPPT), a mathematical model that allows analysts and decision makers to estimate the epidemiologic impact and cost of alternative VMMC scale-up programs. In this study, the researchers use DMPPT to estimate the impact and cost of scaling up adult VMMC in the 13 priority countries in eastern and southern Africa.

**What Did the Researchers Do and Find?:**

The researchers derived VMMC unit costs for each priority country based on a cost assessment undertaken in Zimbabwe, one of the first countries to scale up VMMC services using WHO's “Models for Optimizing Volume and Efficiencies” (MOVE) guidelines. They fed these costs and recent epidemiologic data (including HIV infection rates and the effectiveness of VMMC in preventing HIV transmission) and demographic data (including the adult population size and pre-scale-up male circumcision prevalence) collected in each country into the DMPPT, together with information on the lifetime costs of HIV treatment. Results from running the DMPPT model suggest that scaling up adult VMMC to reach 80% coverage in the 13 priority countries by 2015 would require 20.33 million circumcisions to be completed between 2011 and 2015. To maintain this coverage, a further 8.42 million circumcisions would be required between 2016 and 2025. Such a scale-up would avert 3.36 million new HIV infections through 2025 and would cost US$2,000,000,000 between 2011 and 2025. However, it would result in net savings (because of averted treatment and care costs) of US$16,510,000,000.

**What Do These Findings Mean?:**

These findings suggest that rapid VMMC scale-up in eastern and southern Africa is warranted, given its likely impact on the region's HIV epidemics and the resultant cost savings. However, the accuracy of these findings depends on the assumptions built into the DMPPT and on the data fed into it. For example, there could be risk behavior changes after circumcision. That is, risky sexual behaviors may increase in men who have been circumcised. However, the researchers show that, except in Rwanda, post-circumcision risk behavior change is unlikely to completely reverse the benefits of VMMC. These modeling results also assume that men seeking out VMMC services are typical of the general male population, but if they are actually at unusually low risk of HIV infection, then the benefits of VMMC reported here are likely to be overestimated. Finally, these findings assume 80% VMMC coverage. This may be optimistic, although results from Kenya indicate that this target is achievable. Thus, countries and their international partners must allocate sufficient resources to VMMC scale-up to achieve high coverage rates if they are to take full advantage of the benefits predicted here for VMMC scale-up.

**Additional Information:**

Please access these websites via the online version of this summary at http://dx.doi.org/10.1371/journal.pmed.1001132.

- This study is part of a PLoS Collection of articles on http://www.ploscollections.org/VMMC2011 and is further discussed in a *PLoS Medicine* Review Article by Hankins et al. (http://dx.doi.org/10.1371/journal.pmed.1001127)
- Information is available from WHO, UNAIDS, and PEPFAR on all aspects of HIV/AIDS; the 2011WHO/UNAIDS progress report on VMMC scale-up in the 13 priority countries is available
- NAM/aidsmap provides basic information about HIV/AIDS, summaries of recent research findings on HIV care and treatment, and information on male circumcision for the prevention of HIV transmission
- Information is available from Avert, an international AIDS charity on many aspects of HIV/AIDS, including information on all aspects of HIV prevention, and on HIV/AIDS in Africa (in English and Spanish)
- The Clearinghouse on Male Circumcision, a resource provided by WHO, UNAIDS, and other international bodies, provides information and tools for VMMC policy development and program implementation, including information on the DMPPT and the MOVE guidance
- Personal stories about living with HIV/AIDS are available through Avert, through NAM/aidsmap, and through the charity website Healthtalkonline

## Introduction

Three randomized controlled trials have shown that voluntary medical male circumcision (VMMC) reduces heterosexual HIV acquisition in men by up to 60% [1]–[3]. On the basis of these trial results, the World Health Organization (WHO) and the Joint United Nations Programme on HIV/AIDS (UNAIDS) now recommend that VMMC be offered to heterosexual men in combination with other effective HIV risk reduction interventions in settings with generalized HIV epidemics and where a substantial proportion of men are not circumcised [4].

The long-term population-level impact of implementing and scaling up VMMC services is expected to be considerable in terms of HIV infections averted, as well as net savings associated with the reduced need for treatment, care, and support of infected individuals. Previous model-based studies estimated that VMMC scale-up in countries with generalized HIV epidemics could result in substantial reductions in HIV transmission and prevalence over time among both men and women [3],[5]–[9]. A recent review of these studies concluded that one HIV infection could be averted for every five to 15 VMMCs performed [10]. Modeling studies also have found that expansion of VMMC services produces net savings when compared to lifetime HIV treatment costs [8],[9],[11]. In addition, studies have shown VMMC to be protective against some other sexually transmitted infections (STIs) in both men and women. VMMC has been found to reduce the risk of herpes simplex virus-2 in men [12] and human papillomavirus in men [12]–[15] and their female partners [16], and is associated with a reduction in the risk of genital ulcer disease [3],[17] and genital cancers [18]–[20] in both men and women.

**Figure 1:**
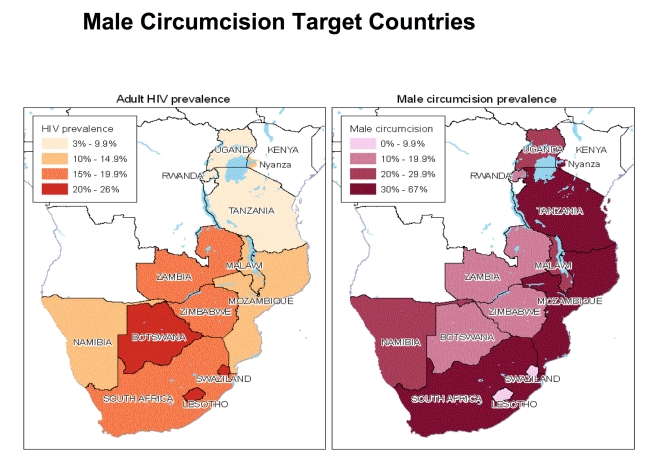
Geographic distribution of HIV and male circumcision prevalence. For the 13 countries included in the study, the map on the left indicates the prevalence of HIV in adults ages 15 to 49 y. The map on the right indicates the percentage of men who are circumcised.

In light of this evidence, in early 2007, WHO and UNAIDS identified the following priority countries for VMMC scale-up: Botswana, Lesotho, Malawi, Mozambique, Namibia, Rwanda, South Africa, Swaziland, Tanzania, Uganda, Zambia, Zimbabwe, and Nyanza Province in Kenya (Figure 1). Four years later, most of these 13 countries have developed plans for VMMC scale-up and are at various stages of program implementation. However, with a few exceptions (e.g., Nyanza Province in Kenya and the Iringa Region in Tanzania), progress in expanding VMMC services remains slow [21].

There is consensus that VMMC scale-up will require substantial funding and massive efforts to adequately train personnel, equip facilities, and ensure the regular distribution of necessary commodities—mostly in settings with weak health systems and resource constraints. As background for the scale-up of safe VMMC, WHO, UNAIDS, and collaborating partners have developed a number of guidelines and toolkits, including a recommended minimum package of VMMC services [22] and clinical guidelines for the provision of these services [23]. In addition, to facilitate a more rapid scale-up of safe VMMC, WHO has outlined considerations for models to optimize the volume and efficiency (MOVE) of VMMC services [24]. Central to these considerations is the efficient use of facility space through the dedication of multiple surgical beds to one surgical team and the coordination of client flow; the efficient use of staff time through task shifting and task sharing, including deployment of non-physicians to complete all or specific steps in VMMC surgery; and the bundling of commodities and supplies required to perform VMMC, including consumable materials and surgical instruments [24].

To support decision making and planning for VMMC scale-up, the United States Agency for International Development (USAID) Health Policy Initiative collaborated with UNAIDS to develop the Decision Makers' Program Planning Tool (DMPPT) [25]. This modeling tool, which has been reviewed by an expert panel [10], allows analysts and decision makers to estimate the epidemiologic impact and cost of alternative programmatic options for scaling up VMMC.

The objective of this study is to estimate the country-specific epidemiologic impact and the cost and net savings associated with scaling up VMMC services based on MOVE considerations in the 13 priority countries in eastern and southern Africa. The perspective that is taken is that of governments and their international partners in their roles as health program funders. To do this, we run country-specific DMPPT models and explore how the results for each country vary by VMMC effectiveness, VMMC coverage level, time to scale-up, level of post-circumcision behavior change, VMMC unit cost, and antiretroviral therapy (ART) cost.

This study expands on previous related work [8],[9],[11] in several ways. All of our analyses are country-specific and based on the most recent data on HIV and male circumcision prevalence available. Our calculations provide more comprehensive estimates of VMMC-related costs than was the case in previous studies since they include costs for supply chain management and waste management, as per WHO's recent recommendations for efficient VMMC scale-up [24]. In addition, we provide country-specific results on impact (VMMC per HIV infection averted), cost-effectiveness (cost per HIV infection averted) and cost savings (cost of VMMC relative to the averted cost of lifetime provision of ART).

## Methods

### The Model

The DMPPT is a Microsoft-Excel-based modeling tool that estimates the epidemiologic impact (HIV infections averted) and the cost and net savings associated with different programming scenarios for VMMC scale-up. A list of all the equations used in the model can be found in Table S1. Additional details, including a copy of the workbook and the model manual [25], can be found at http://www.malecircumcision.org/programs/DMPPT.html.

The DMPPT is a compartmental deterministic model in which the population is disaggregated into four sex/age groups (females ages 15–24 y, males ages 15–24 y, females ages 25–49 y, and males ages 25–49 y). For each population group, a susceptible and infected population is described [26],[27]. The susceptible population is increased by people aging into it (15-y-olds entering the younger age group and 25-y-olds leaving the younger age group and entering the older age group) and decreased by non-AIDS deaths and new infections. The infected population is increased by new infections and decreased by non-AIDS deaths, AIDS deaths, and aging out.

In the model, HIV incidence (the proportion of the susceptible population becoming infected each year) is the product of HIV prevalence in the population and the force of infection. The force of infection is determined by the base rate of infection (which is a fitting parameter specific to each population group), changes in behavior, and changes due to male circumcision. Changes in behavior are assumed to occur as the epidemic progresses because of two key influences. Individuals with the riskiest behaviors are assumed to become infected first and die sooner than the rest of the population. Also, as AIDS deaths accumulate, a powerful effect on individual behavior is assumed, as those who know someone who has died from AIDS are motivated to adopt safer behaviors. Thus, the force of infection can drop over time as the cumulative number of AIDS deaths increases. The amount of the effect is determined by a fitting parameter that produces the best fit to the historical HIV prevalence trend. The impact of other prevention interventions is incorporated through exogenous reductions in the force of infection. The effect of male circumcision on the force of infection is simply the increase in the prevalence of male circumcision multiplied by the reduction in susceptibility due to circumcision.

For each scenario of interest, the DMPPT model generates two projections: a baseline projection, in which male circumcision coverage is held constant at the pre-scale-up level, and a scale-up projection where VMMC coverage is scaled up to the desired coverage level. In the baseline projection, new infections increase over time in response to the initial force of infection. This causes HIV prevalence to rise rapidly in early years. The force of infection then declines somewhat as AIDS deaths accumulate and prevalence stabilizes. Implementation of a male circumcision program can cause a further reduction in the force of infection (depending on the increase in male circumcision coverage), leading to a decline in new infections and HIV prevalence. There is some concern that men who undergo circumcision may develop a false sense of protection against HIV and thereafter decrease or even stop previously implemented protective behaviors [28],[29]. Post-circumcision behavior change is modeled in the DMPPT as a proportional change in the force of infection, with a VMMC scale-up scenario in which circumcision does not lead to any behavior change being modeled as having a 0% post-circumcision behavior change effect.

The DMPPT model calculates the yearly cost of the additional circumcisions (the number of VMMCs required above and beyond those occurring at pre-scale-up coverage levels) as the sum of the additional circumcisions performed per annum times the VMMC unit cost. The model also calculates the net savings associated with HIV infections averted and the subsequent treatment costs averted.

### Demographic, Epidemiologic, and Sexual Behavior Data

The demographic and epidemiologic inputs required to estimate the epidemiologic impact of VMMC scale-up include the following:

- Demographic data—size of adult population by sex and age group, adult population growth rate, crude birth rate, crude death rate, proportion of population surviving to age 15 y, pre-scale-up male circumcision prevalence.
- Epidemiologic data—HIV prevalence among adults by sex and age group; effectiveness of male circumcision in preventing HIV infection; underlying HIV transmission factors based on scientific evidence, including probability of transmission from mother to child; fertility reduction due to HIV.
- Sexual behavior data—sexual mixing patterns by sex and age group, post-circumcision behavior change (change in condom use and/or change in number of sexual partners following VMMC resulting from lowered perceived risk).

We obtained the required demographic, epidemiologic, and sexual behavior data for each country from recent household surveys [30]–[42] and Spectrum, a modeling software package, which includes a demographic platform populated with UN population data [27],[43],[44]. Country-specific pre-scale-up population characteristics, including size of the adult population, adult population growth rate, male circumcision and adult HIV prevalence, and HIV incidence, are shown in Table 1. A complete listing of the data used for each country is available from the authors upon request.

**Table 1:** Pre-scale-up population characteristics, by country.

Country	Population Aged 15–49 y (Millions)	Adult Population Growth Rate (Percentage)	Male Circumcision Prevalence (Percentage)	Adult HIV Prevalence (Percentage)	Adult HIV Incidence (Percentage)
Botswana	1.08	2.42	10.2	22.9	2.0
Lesotho	1.06	1.34	0.0	23.6	2.9
Malawi	6.84	3.02	20.7	12.5	1.0
Mozambique	10.32	2.45	59.5	12.9	1.4
Namibia	1.09	2.22	21.0	14.1	0.8
Nyanza Province, Kenya	1.51	2.80	44.8	13.1	1.6
Rwanda	4.49	3.29	10.0	3.1	0.3
South Africa	26.84	0.89	44.7	16.9	2.1
Swaziland	0.59	1.51	8.2	26.4	3.2
Tanzania	19.63	2.89	66.8	6.7	0.8
Uganda	14.16	3.87	24.8	6.5	0.7
Zambia	6.85	2.61	10.8	14.9	1.6
Zimbabwe	6.19	1.50	10.3	17.9	2.2

Table 1 provides the size of the 2008 population aged 15–49 y, adult population growth rate, and adult HIV incidence from Spectrum, which is populated with UN population data [27],[43],[44]. The years for the pre-scale-up male circumcision and adult HIV prevalence data are as follows: Botswana (2004) [30], Lesotho (2004) [9],[39], Malawi (2004) [40], Mozambique (2003) [35], Namibia (2007) [38], Nyanza Province, Kenya (2008/2009) [36], Rwanda (2005) [9],[34], South Africa (2008), Swaziland (2006/2007) [32], Tanzania (2007/2008) [42], Uganda (2004) [37], Zambia (2007) [31], and Zimbabwe (2005/2006) [33].

### Cost Data

A breakdown of the VMMC unit cost used in the model is provided in Table 2. With the exception of the waste management and supply chain costs, the unit cost components are derived from a VMMC costing study conducted in Zimbabwe in 2010 as part of a multi-country study that also included Kenya, Namibia, South Africa, Uganda, and Zambia [45]–[50]. Zimbabwe was chosen because it is one of the first countries to scale up VMMC services following WHO's MOVE considerations for more efficient use of facility space and staff time and for the bundling of the commodities required to perform VMMC [24]. Information on required inputs and costs was collected from sites providing VMMC in Zimbabwe. This includes data on both direct costs (consumables, staff costs, and training costs) and indirect costs (capital costs, maintenance and utility costs, support overhead, and management overhead). Because significant variations in labor costs were noted across countries, we vary the staff costs by country, adjusting them by after tax median monthly disposable salary. We also adjust the costing information collected in the Zimbabwe study for underreporting on waste management and other supply chain costs [51]. We base the waste management costs on a costing analysis conducted in Swaziland, a detailed description of which is provided elsewhere [52]. The VMMC unit cost used for each country is shown in Table 3.

**Table 2:** VMMC unit cost components.

Category	Cost Component	Cost (US$)
**Direct costs**	Consumables	28.67
	Waste management	9.39
	Other supply chain	10.91
	Staff costs	Variable^a^
	Training costs	1.70
**Indirect costs**	Capital costs	0.45
	Maintenance and utility costs	3.24
	Support overhead	4.42
	Management overhead	2.32

Table 2 presents the components of the VMMC unit cost used in this study. The VMMC unit cost was derived based on a VMMC costing study conducted in Zimbabwe in 2010 [50] and supplemented with analysis of data from Swaziland.

a We vary staff costs by country, adjusting them by after tax median monthly disposable salary.

**Table 3:** VMMC unit cost by country.

Country	VMMC Unit Cost—Base Case (US$)	VMMC Unit Cost Range (US$)
Botswana	78.07	62.45–93.68
Lesotho	83.78	67.03–100.54
Malawi	83.78	67.03–100.54
Mozambique	86.29	69.03–103.55
Namibia	86.60	69.28–103.92
Nyanza Province, Kenya	74.89	59.92–89.87
Rwanda	80.13	64.10–96.16
South Africa	95.15	76.12–114.18
Swaziland	74.83	59.86–89.79
Tanzania	82.56	66.04–99.07
Uganda	65.85	52.68–79.02
Zambia	89.65	71.72–107.58
Zimbabwe	78.23	62.58–93.87

Table 3 shows the country-specific VMMC unit costs used in the study. To obtain the VMMC unit cost for each country, we vary staff costs in each country, adjusting them by after tax median monthly disposable salary [51]. The range used in the sensitivity analysis is country base case unit cost ±20%.

For lifetime HIV treatment costs, we use the default discounted value in the DMPPT model (US$7,400) [25]; we use the same value for all countries. This value includes costs of AIDS treatment and care, including ART, treatment of major opportunistic infections, laboratory tests, and home-based care. The lifetime HIV treatment cost is based on a unit cost of US$155 for first-line antiretroviral drugs and US$1,678 for second-line antiretroviral drugs [53]. We also assume that need for treatment begins after 8 y of infection and that the annual continuation rate on ART is 97.5% [27].

Following common practice in economic evaluation, we apply an annual discount rate of 3% on future expenditures and savings [54] as well as on infections averted [55].

### Base Case and Sensitivity Analysis

Our analyses are limited to the scale-up of VMMC services among males ages 15 to 49 y who are HIV-negative. For all models, we assume that the scale-up begins in 2011 and that the pace of scale-up is slower at first, followed by a more rapid scale-up, and then another slow period of scale-up, to allow for training and other logistic developments likely to occur in the early stages of implementation. The model observation period for all models is 2011 to 2025. This allows us to evaluate the 15-y impact of VMMC more comprehensively.

For the base case, we assume the following:

- VMMC effectiveness—we use 60% for the protective effect of VMMC and assume that the effect is constant over the model simulation period [1],[56].
- Target VMMC coverage level—male circumcision coverage is increased from pre-scale-up levels to 80% of HIV-negative males ages 15 to 49 y.
- Time to 80% scale-up—male circumcision coverage is increased from pre-scale-up levels to 80% of HIV-negative males ages 15 to 49 in 5 y, with the coverage target reached by 2015 and maintained at 80% thereafter.
- Post-circumcision behavior change—no post-circumcision behavior change is associated with VMMC scale-up.
- VMMC unit cost—the VMMC unit cost used for each country is shown in Table 3.

Given uncertainty in model inputs, we also conduct sensitivity analyses, exploring various alternative scenarios. We vary VMMC effectiveness, VMMC coverage level, time to scale-up, level of post-circumcision behavior change, VMMC unit cost, and lifetime HIV treatment cost as follows:

- VMMC effectiveness—34% and 77% (versus 60%) [1].
- Target VMMC coverage levels—50% and 100% (versus. 80%).
- Time to 80% scale-up—1, 10, and 15 y (versus 5 y).
- Post-circumcision behavior change—a 30% post-circumcision behavior change effect is associated with MMC scale-up (versus no post-circumcision behavior change, or 0%).
- VMMC unit cost—country base case unit cost ±20% as shown in Table 3.
- Lifetime ART cost—ART cost is assumed to decline over time, leading to a lifetime cost of US$3,400 (versus US$7,400) [53].

## Results

### Base Case—80% VMMC Coverage by 2015

The number of additional VMMCs required to achieve 80% male circumcision coverage by 2015 is presented in Table 4. A total of 20.34 million VMMCs are required to scale up male circumcision coverage to 80% by 2015 in Botswana, Lesotho, Malawi, Mozambique, Namibia, Rwanda, South Africa, Swaziland, Tanzania, Uganda, Zambia, Zimbabwe, and Nyanza Province in Kenya. When taking the longer-term view (to 2025), an additional 8.42 million VMMCs are required between 2016 and 2025, for a total of almost 29 million VMMCs in the model's full study period (2011–2025). The number of VMMCs needed to achieve 80% male circumcision by 2015 varies by country, with the largest number of VMMCs required in South Africa (the country with the largest population and a high adult HIV prevalence) followed by Uganda (the country with a large and fast-growing population and low baseline VMMC prevalence), Malawi, Zambia, and Zimbabwe (also countries low baseline VMMC prevalence).

**Table 4:** Impact of VMMC on HIV infections averted in base case, by country, 2011–2015 and 2011–2025.

Country	Time Period	Additional VMMCs (Millions)	HIV Infections Averted (Millions)	HIV Infections Averted (Percentage)	VMMC per HIV Infection Averted
**Botswana**	2011–2015	0.35	0.01	12	36
	2011–2025	0.49	0.06	28	8
**Lesotho**	2011–2015	0.38	0.01	12	28
	2011–2025	0.54	0.11	37	5
**Malawi**	2011–2015	2.10	0.03	11	66
	2011–2025	3.04	0.24	28	13
**Mozambique**	2011–2015	1.06	0.03	5	38
	2011–2025	1.53	0.22	13	7
**Namibia**	2011–2015	0.33	0.00	9	109
	2011–2025	0.48	0.02	25	26
**Nyanza Province, Kenya**	2011–2015	0.38	0.01	6	36
	2011–2025	0.57	0.07	16	8
**Rwanda**	2011–2015	1.75	0.01	10	239
	2011–2025	2.53	0.06	29	44
**South Africa**	2011–2015	4.33	0.14	7	30
	2011–2025	5.94	1.08	20	5
**Swaziland**	2011–2015	0.18	0.01	12	25
	2011–2025	0.27	0.06	34	5
**Tanzania**	2011–2015	1.38	0.02	3	56
	2011–2025	1.98	0.20	9	10
**Uganda**	2011–2015	4.25	0.05	10	91
	2011–2025	6.35	0.34	25	19
**Zambia**	2011–2015	1.95	0.04	12	43
	2011–2025	2.87	0.34	30	8
**Zimbabwe**	2011–2015	1.91	0.06	13	31
	2011–2025	2.17	0.57	42	4
**Total**	2011–2015	20.34	0.43	8	47
	2011–2025	28.76	3.36	22	9

Table 4 presents the additional VMMCs and the impact of VMMC on HIV infections averted for 2011–2015 and 2011–2025 for the base case scenario. HIV infections averted are not discounted.

We find a strong impact of scaling up VMMC to achieve 80% coverage by 2015 on the number of adult HIV infections averted. A total of 430,000 HIV infections are averted in the 13 countries between 2011 and 2015, while almost 3.36 million HIV infections are averted by 2025 (Table 4). South Africa is the country with the largest number of HIV infections averted (with more than 1 million infections averted between 2011 and 2025) followed by Zimbabwe, Zambia, and Uganda. More than 20% of new HIV infections are averted between 2011 and 2025 in all countries except Mozambique, Nyanza Province (Kenya), South Africa, and Tanzania (the countries with the highest baseline VMMC prevalence). Zimbabwe is the country with the highest percentage of new HIV infections averted, with 42% of new infections averted between 2011 and 2025. The large benefit of VMMC in Zimbabwe is largely driven by the currently low prevalence of VMMC (10.3%) and high HIV prevalence (17.9%) and incidence (2.2%).

The number of VMMCs required to avert one HIV infection is calculated by dividing the additional number of VMMCs required by the number of HIV infections averted over the relevant time period. For the period 2011–2015, the number of VMMCs per HIV infection averted ranges from 25 in Swaziland to 239 in Rwanda, while for the full study period (2011–2025), the number of VMMCs per HIV infection averted ranges from four in Zimbabwe to 44 in Rwanda (Table 4). For the period 2011–2025, the number of VMMCs per HIV infection averted is ten or less in all countries except Malawi, Namibia, Rwanda, and Uganda, each of which has a relatively low incidence of HIV.

**Figure 2:**
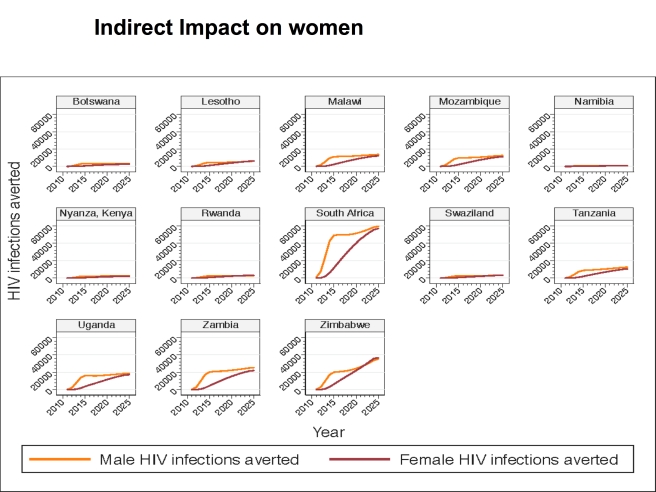
HIV infections averted in base case, by male and female, by country, 2011–2025. Figure 2 provides data for males and females for 2011–2025 for the base case scenario in which 80% coverage of VMMC is reached in 2015 and maintained thereafter, and there is no post-circumcision risk behavior change.

Although circumcision of HIV-infected men has not been found to directly reduce HIV transmission to their female partners [57], and the primary impact of increasing VMMC coverage is to reduce the number of new HIV infections in men, the number of new infections in women is also reduced. This occurs by reducing the exposure of women to HIV-infected men. That is, as HIV incidence decreases in men following VMMC scale-up, the probability of women encountering infected male partners decreases, with a consequent reduction in HIV incidence among women. The HIV infections averted (presented in Table 4) thus represent infections averted in both men and women. Figure 2 illustrates the male and female HIV infections averted over time, by country, for 2011–2025. In all countries, the cumulative number of male HIV infections averted between 2011 and 2025 is higher than the cumulative number of female HIV infections averted. In the early years, the HIV infections averted occur mostly among men, but over time, the proportion of HIV infections averted in women steadily increases, with new HIV infections averted in women representing almost half of the total HIV infections averted in 2025.

**Figure 3:**
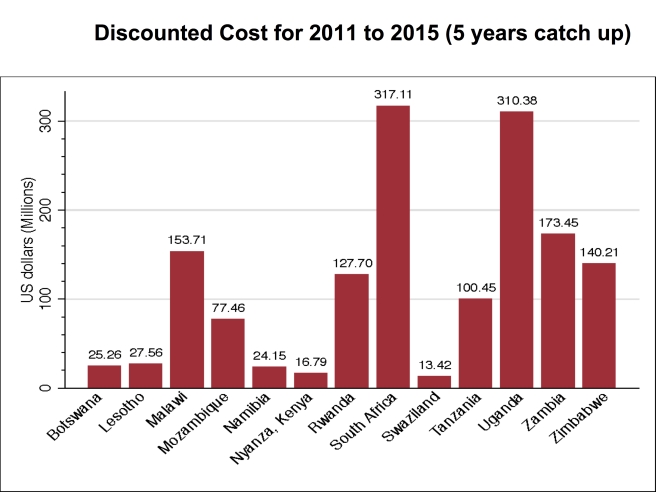
Cost for scaling up VMMC coverage in base case, by country, 2011–2015. Figure 3 provides the discounted cost of scaling up VMMC coverage for 2011–2015 for the base case scenario in which 80% coverage of VMMC is reached in 2015 and maintained thereafter, and there is no post-circumcision risk behavior change.

Figure 3 shows the discounted cost of scaling up VMMC to achieve 80% coverage by 2015 for each of the 13 priority countries for the period 2011–2015. The cost ranges from US$12.53 million in Swaziland (the country with the smallest number of additional VMMCs required) to US$376.55 million in South Africa (the country with the highest number of additional VMMCs required), with a total of US$1,520,000,000 needed to scale up VMMC coverage to 80% in the 13 countries by 2015. The cost for the period 2011–2025 is shown in the first column of Table 5. A total of over US$2,000,000,000 is needed to scale up VMMC coverage to 80% by 2015 and maintain this level of coverage in the 13 countries until 2025.

**Table 5:** Total cost and net savings per HIV infection averted for base case scenario, by country, 2011–2025.

Country	Cost of VMMC Scale-Up, 2011–2025 (US$ Millions)	Cost per HIV Infection Averted, 2011–2025 (US$)	Net Savings, 2011–2025 (US$ Millions)	Net Savings per HIV Infection Averted, 2011–2025 (US$)
Botswana	32.79	693	317.14	6,707
Lesotho	34.97	442	550.57	6,958
Malawi	218.46	1,216	1,110.98	6,184
Mozambique	113.32	704	1,077.25	6,696
Namibia	35.52	2,558	67.21	4,842
Nyanza Province, Kenya	36.38	660	371.23	6,740
Rwanda	173.78	4,096	140.21	3,304
South Africa	489.47	605	5,498.55	6,795
Swaziland	17.18	406	295.88	6,994
Tanzania	140.40	950	953.74	6,450
Uganda	357.29	1,408	1,520.36	5,992
Zambia	220.42	869	1,679.41	6,623
Zimbabwe	153.84	369	2,930.64	7,031
Total	2,023.80	809	16,513.15	6,608

Table 5 provides the discounted cost of scaling up VMMC coverage, cost per HIV infection averted, and net savings per HIV infection averted for 2011–2025 for the base case scenario. HIV infections averted are discounted.

Combining the cost data with the number of HIV infections averted, we obtain the discounted cost per HIV infection averted. The cost per HIV infection averted for the period 2011–2025 ranges from US$369 in Zimbabwe, where adult HIV prevalence is 17.9%, to US$4,096 in Rwanda, where adult HIV prevalence is lower than in any of the other 13 countries studied (3.1%). The overall cost per HIV infection averted for 2011–2025 for all 13 countries is US$809.

Finally, we calculate the net savings associated with VMMC scale-up. The savings due to future ART costs avoided (with the discounted value of lifetime HIV treatment costs estimated at US$7,400 per infection) minus the discounted VMMC costs amount to US$16,510,000,000 for the 13 countries from 2011 to 2025 (Table 5). The net savings can also be combined with the number of HIV infections averted to obtain the net savings per HIV infection averted. For 2011–2025, this value ranges from US$3,304 in Rwanda to US$7,031 in Zimbabwe. The overall net savings per HIV infection averted for 2011–2025 for all 13 countries is US$6,608.

### Sensitivity Analysis

Table 6 presents the results of the sensitivity analysis. The table provides the values for the HIV infections averted, the number of VMMCs per HIV infection averted, the cost per HIV infection averted, and the net savings per HIV infection averted for 2011–2025 for each parameter examined. The values for the base scenario (80% male circumcision coverage within 5 y) are shown in the third column of Table 6.

**Table 6:** Sensitivity analysis of key parameters (2011–2025).

Country	Results from DMPPT	Base Case Scenario	VMMC Effectiveness		VMMC Coverage		Time to Scale-Up			BC 30%^a^	Cost per VMMC		ART Cost = US$3,400
			34%	77%	50%	100%	1 y	10 y	15 y		−20% Base Case Cost	+20% Base Case Cost	
**Botswana**	IA	62,773	34,504	82,029	35,620	80,877	75,952	45,226	30,171	62,773	62,773	62,773	62,773
	VMMC per IA	8	14	6	8	8	7	10	14	8	8	8	8
	Cost per IA (US$)	693	1,253	533	694	694	605	881	1,197	836	555	832	693
	Net savings per IA (US$)	6,707	6,147	6,867	6,706	6,706	6,795	6,519	6,203	6,564	6,845	6,568	2,707
**Lesotho**	IA	106,427	60,123	132,245	66,385	129,578	130,568	73,664	46,994	100,848	106,427	106,427	106,427
	VMMC per IA	5	9	4	5	5	4	7	10	5	5	5	5
	Cost per IA (US$)	442	777	356	440	454	382	579	816	466	354	530	442
	Net savings per IA (US$)	6,958	6,623	7,044	6,960	6,946	7,018	6,821	6,584	6,934	7,046	6,870	2,958
**Malawi**	IA	240,685	131,912	315,299	121,012	317,639	292,121	172,169	113,722	172,576	240,685	240,685	240,685
	VMMC per IA	13	23	10	12	13	11	16	23	18	13	13	13
	Cost per IA (US$)	1,216	2,210	930	1,194	1,233	1,064	1,543	2,100	1,696	973	1,459	1,216
	Net savings per IA (US$)	6,184	5,190	6,470	6,206	6,167	6,336	5,857	5,300	5,704	6,427	5,941	2,184
**Mozambique** b	IA	215,861	103,714	314,141		421,592	262,897	153,291	100,400	159,030	215,861	215,861	215,861
	VMMC per IA	7	14	5		7	6	9	13	10	7	7	7
	Cost per IA (US$)	704	1,440	491		714	615	900	1,235	954	563	845	704
	Net savings per IA (US$)	6,696	5,960	6,909		6,686	6,785	6,500	6,165	6,446	6,837	6,555	2,696
**Namibia**	IA	18,373	9,936	24,335	9,243	24,174	23,160	12,520	7,924	12,947	18,373	18,373	18,373
	VMMC per IA	26	48	20	25	26	21	36	53	37	26	26	26
	Cost per IA (US$)	2,558	4,741	1,929	2,502	2,601	2,134	3,453	4,955	3,629	2,047	3,070	2,558
	Net savings per IA (US$)	4,842	2,659	5,471	4,898	4,799	5,266	3,947	2,445	3,771	5,353	4,330	842
**Nyanza Province, Kenya**	IA	73,420	35,776	105,322	2,382	72,961	53,834	32,309	21,649	31,567	44,636	44,636	73,420
	VMMC per IA	8	16	5	8	8	7	10	13	11	8	8	8
	Cost per IA (US$)	660	1,337	465	661	661	585	820	1,089	933	528	793	660
	Net savings per IA (US$)	6,740	6,063	6,935	6,739	6,739	6,815	6,580	6,311	6,467	6,872	6,607	2,740
**Rwanda** c	IA	56,840	32,138	72,988	33,163	72,039	68,999	40,514	26,480	(1,183)	56,840	56,840	56,840
	VMMC per IA	44	79	35	43	45	38	58	81	(2,132)	44	44	44
	Cost per IA (US$)	4,096	7,248	3,188	4,015	4,152	3,594	5,195	7,118	(192,148)	3,276	4,915	4,096
	Net savings per IA (US$)	3,304	152	4,212	3,385	3,248	3,806	2,205	282	N.A.	4,124	2,485	(696)
**South Africa**	IA	1,083,869	553,267	1,496,933	164,840	1,677,803	1,340,315	750,846	478,788	954,426	1,083,869	1,083,869	1,083,869
	VMMC per IA	5	11	4	5	6	5	7	11	6	5	5	5
	Cost per IA (US$)	605	1,161	444	595	614	518	798	1,135	685	484	726	605
	Net savings per IA (US$)	6,795	6,239	6,956	6,805	6,786	6,882	6,602	6,265	6,715	6,916	6,674	2,795
**Swaziland**	IA	56,810	31,886	71,520	33,356	70,597	69,466	39,535	25,032	53,234	56,810	56,810	56,810
	VMMC per IA	5	8	4	5	5	4	6	9	5	5	5	5
	Cost per IA (US$)	406	717	323	401	418	366	534	755	436	325	487	406
	Net savings per IA (US$)	6,994	6,683	7,077	6,999	6,982	7,034	6,866	6,645	6,964	7,075	6,913	2,994
**Tanzania** b	IA	199,249	92,261	300,691		492,563	241,090	143,379	95,386	173,793	199,249	199,249	199,249
	VMMC per IA	10	21	7		10	9	13	18	11	10	10	10
	Cost per IA (US$)	950	2,035	633		967	838	1,190	1,600	1,090	760	1,139	950
	Net savings per IA (US$)	6,450	5,365	6,767		6,433	6,562	6,210	5,800	6,310	6,640	6,261	2,450
**Uganda**	IA	339,524	182,516	451,568	157,211	458,128	408,009	245,533	1,654,578	104,102	339,524	339,524	339,524
	VMMC per IA	19	35	14	18	19	16	24	32	61	19	19	19
	Cost per IA (US$)	1,408	2,615	1,060	1,387	1,422	1,250	1,755	2,322	4,589	1,126	1,690	1,408
	Net savings per IA (US$)	5,992	4,785	6,340	6,013	5,978	6,150	5,645	5,078	2,811	6,274	5,710	1,992
**Zambia**	IA	339,632	188,110	441,327	252,260	570,773	536,129	321,756	215,692	407,557	444,352	444,352	339,632
	VMMC per IA	8	15	7	8	8	7	10	14	9	8	8	8
	Cost per IA (US$)	869	1,560	671	865	874	769	1,082	1,440	948	695	1,043	869
	Net savings per IA (US$)	6,531	5,840	6,729	6,535	6,526	6,631	6,318	5,960	6,452	6,705	6,357	2,531
**Zimbabwe**	IA	565,749	311,075	691,348	320,894	686,019	699,894	375,010	231,007	520,864	565,751	565,751	565,749
	VMMC per IA	4	7	3	4	4	3	6	9	4	4	4	4
	Cost per IA (US$)	369	669	300	370	390	309	523	795	401	292	437	369
	Net savings per IA (US$)	7,031	6,731	7,100	7,030	7,010	7,091	6,877	6,605	6,999	7,108	6,963	3,031

Table 6 presents sensitivity analysis for the following key parameters: VMMC effectiveness, VMMC coverage, time to scale-up, level of post-circumcision behavior change, VMMC unit cost, and lifetime HIV treatment cost.

a BC is post-circumcision risk behavior change.

b No models were run for the scale-up to 50% VMMC coverage scenario for Mozambique and Tanzania, since national pre-scale-up VMMC coverage in these two countries was greater than 50%.

c Scaling up VMMC in the presence of 30% post-circumcision risk behavior change in Rwanda resulted in an increase in HIV infections.

IA, infection(s) averted.

The results help to confirm the internal consistency of the DMPPT model and show that, in large measure, the VMMC impact, cost, and savings results that we observe in the base case are robust to changes in VMMC effectiveness, VMMC coverage, time to scale-up, level of post-circumcision behavior change, VMMC unit cost, and lifetime HIV treatment cost.

Our analysis varying VMMC effectiveness shows that even if we assume much reduced effectiveness (34% instead of 60%), high numbers of infections are averted and net savings per infection averted are obtained. The same is true if the VMMC coverage target is reduced from 80% to 50% or if the time to achieve 80% VMMC coverage is increased to 10 or 15 y.

To assess the potential impact of post-circumcision risk behavior change, the model calculates the impact of risky sexual behaviors reverting to patterns that existed earlier in the epidemic, prior to the scale-up of VMMC services. It should be noted that this impact is different from the early resumption of sexual activity before complete wound healing that can also be an issue of concern in the context of expanding VMMC services. The impact is the result of behavior change due to a false perception that VMMC has eliminated the risk of HIV transmission. As compared to no post-circumcision risk behavior change, assuming 30% post-circumcision risk behavior change results in a decrease in the HIV infections averted, an increase in the numbers of VMMCs per HIV infection averted, an increase in the cost per HIV infection averted, and a decrease in net savings. In all of the countries studied except Rwanda, VMMC scale-up remains cost saving, even with 30% post-circumcision risk behavior change. In Rwanda, the country with the lowest HIV prevalence, scaling up VMMC in the presence of this level of post-circumcision risk behavior leads to an increase in the number of new HIV infections.

Predictably, decreasing (increasing) the base case VMMC unit cost by 20% results in a decrease (increase) in the cost per HIV infection averted and an increase (decrease) in the net savings per HIV infection averted in countries. Assuming the lower VMMC unit cost for each country, the cost and net savings associated with scaling up VMMC coverage to 80% by 2015 and maintaining this level of coverage in the 13 countries until 2025 are US$1,620,000,000 and US$16,900,000,000 instead of US$2,020,000,000 and US$16,510,000,000 in the base case. Assuming the higher VMMC unit cost, the cost of scaling up VMMC services in the 13 countries is US$2,430,000,000, resulting in net savings of US$16,090,000,000.

Reducing lifetime ART cost from US$7,400 to US$3,400 does significantly reduce the net savings per infection averted in all countries, although it remains well above US$2,000 in almost all countries. Rwanda is the only country where such a reduction in lifetime ART cost would lead to net losses per infection averted. Net savings associated with scaling up VMMC coverage is reduced to US$6,480,000,000 in the 13 countries if lifetime ART cost is US$3,400.

## Discussion

To support decision making and planning for VMMC scale-up in Botswana, Lesotho, Malawi, Mozambique, Namibia, Rwanda, South Africa, Swaziland, Tanzania, Uganda, Zambia, Zimbabwe, and Nyanza Province in Kenya, we estimate the country-specific impact and cost of scaling up VMMC services using the DMPPT model developed by the USAID and UNAIDS. Our study suggests that rapid scale-up of VMMC in eastern and southern Africa is warranted based on the likely impact on the region's HIV epidemics and the resultant cost savings.

Model results also show that, although the primary impact of scaling up VMMC coverage is to reduce the number of new HIV infections in men, the intervention also prevents HIV infections in women. While the cumulative number of male HIV infections averted between 2011 and 2025 is higher than the cumulative number of female HIV infections averted, the proportion of HIV infections averted in women steadily increases until HIV infections averted in women represent almost half of the new HIV infections averted in 2025.

Our results show that the costs per infection averted are comparable to the estimated cost per infection averted of a number of key HIV prevention interventions implemented in the region. The cost per HIV infection averted, which ranges from US$369 in Zimbabwe to US$4,096 in Rwanda, is below US$1,000 in all countries except Malawi, Namibia, Rwanda, and Uganda. These costs per infection averted are comparable to those of prevention of vertical transmission (US$663 per HIV-positive birth averted), voluntary counseling and testing (US$1,315 per HIV infection averted), and prevention of STIs (US$321–US$1,665) [58].

Our results are somewhat sensitive to changes in VMMC effectiveness, VMMC coverage targets, time to scale-up, and VMMC cost. Nonetheless, all of the values tested for these parameters resulted in lives saved and cost savings for all countries. It is especially important to note that even if VMMC coverage is scaled up to 50% in 5 y instead of to 80%, high numbers of infections will be averted and net savings per infection averted will be obtained in all countries except Mozambique and Tanzania—the two countries where pre-scale-up coverage is higher than 50%. This is critical because even though there is no evidence available yet on the feasibility of scaling up VMMC coverage to 80%, there is evidence that VMMC coverage rates of above 50% can be reached through VMMC scale-up efforts [56].

Similar to the results reported by other authors, our results suggest that post-circumcision risk behavior change is unlikely to completely reverse the benefits of male circumcision, with the possible exception of Rwanda. In the case of Rwanda, the low incidence of HIV (0.3%) means that risk compensation associated with male circumcision could actually make the epidemic worse. In the remaining countries, the negative consequences from a 30% risk compensation would be insufficient to fully reverse the benefits of VMMC. In all countries, it is critical that VMMC programs emphasize that male circumcision is not 100% protective and that condom use and other behavioral risk reductions remain essential [8],[59]. Effective messaging in this regard is critical to ensure that men and their sexual partners do not increase their sexual risk behaviors following VMMC scale-up.

This study has a number of limitations. Because this model was designed primarily for advocacy purposes, certain elements are not modeled, including treatment, a more fully articulated age structure, and force of infection, which probably leads to an overestimate of the impact of male circumcision.

Another limitation is that we use pre-scale-up male circumcision coverage estimates based on men's self-reported circumcision status in household surveys. There is some evidence that uncircumcised and partially circumcised men may report being circumcised in some populations [60]. Unfortunately, there is no information available on the prevalence of this phenomenon in the countries studied. In the one country where partial circumcision is known to be widely practiced (Lesotho), we assume baseline VMMC prevalence to be at 0% rather than using the self-reported data.

Additional limitations relate to our cost assumptions. Ideally, we would have obtained country-specific VMMC unit cost estimates based on the MOVE model instead of only adjusting the staff costs by country based on after-tax median monthly disposable salary. However, this model for delivering VMMC was available in only a limited number of sites. Our VMMC unit cost, which is higher than that used in previous studies [8],[11],[59],[61],[62], reflects a better understanding of the service delivery components required for such a program, including waste management and logistics associated with the scaling up of VMMC services [52]. It should also be noted that countries are procuring the same circumcision kits and are adopting the same service delivery model that was costed in this study. If possible, we would also have adjusted VMMC unit cost over time and scale [63],[64]. However, no information is currently available on how VMMC unit cost might vary over time and with scale. Another limitation is that we do not include costs associated with demand creation in our study [65]. Finally, it would have been preferable for us to obtain country-specific ART costs; however, country-specific data for the 13 countries studied were not available.

HIV prevalence and incidence estimates used in the model and how they compare to the actual present and projected prevalence and incidence in each of the selected countries is another factor that affects the accuracy of our findings. A steeper decline in baseline incidence, for example, will result in fewer infections averted as a result of VMMC. Conversely, if these incidence trends are overly optimistic, then the number of infections available to be averted by VMMC may in turn be larger than estimated here.

We also recognize that our modeling assumes that males seeking out VMMC services are typical of the general male population in the selected age range. If, for example, those seeking out VMMC services are in fact those who are at least risk of becoming infected (perhaps VMMC clients already disproportionately use condoms), then the benefits of VMMC are likely to be overestimated. Conversely, if those who are at most risk of infection (those with a large number of partners and/or low condom use) are disproportionately attracted to VMMC services, then the modeling presented in this paper may underestimate the value of VMMC scale-up. Given that VMMC client sexual behavior, relative to the behavior of the general male population, remains unknown, it is not possible to determine whether our projections are underestimated or overestimated.

There are also a number of prevention benefits that are indirectly associated with seeking out VMMC services. These include the benefits of counseling and testing, STI treatment, and early antiretroviral treatment. For example, for those males who seek out VMMC services but find that they are already HIV-infected, there could be societal prevention benefits if these men subsequently are able to access early treatment and reduce their infectivity. Since we focus only on the direct benefits received by those receiving VMMC and do not quantify any additional benefits of counseling and testing, STI treatment, and early antiretroviral treatment as a result of seeking VMMC services, it is possible that the prevention benefits of VMMC could be underestimated.

The past year has been marked by important advances in HIV prevention, including the finding that early treatment initiation reduces sexual transmission by as much as 96% in stable serodiscordant couples [66]. However, the population-level impact of treatment is not currently well known. And coverage of those eligible for antiretroviral treatment under WHO guidelines remains low, with less than half of people needing treatment having access to it in eastern and southern Africa in 2010 [67].

VMMC is a one-time surgical procedure with the promise of substantial impact on population-level HIV incidence. Its effects accrue to both men and women, and it compares favorably with other HIV prevention programs in terms of cost-effectiveness. Countries are adopting WHO/UNAIDS recommendations to incorporate VMMC within their HIV prevention portfolios. Best practices from Kenya [68], Tanzania [69], and South Africa [56] show that with government leadership, community involvement, and collaboration among partners, it is possible to scale up rapidly by implementing service delivery models that take into consideration available resources, efficiency, and quality to achieve the maximum public health impact. To ensure that high coverage rates are achievable, countries and their international partners should allocate enough resources to take advantage of the returns on investment predicted for this biomedical HIV prevention modality.

## Supporting Information

Table S1 **Model details and equations.**
(DOCX)

## Abbreviations

ART: antiretroviral therapy
CDC, Centers for Disease Control and Prevention; DMPPT: Decision Makers' Program Planning Tool
MOVE: Models for Optimizing Volume and Efficiencies
PEPFAR, United States President's Emergency Plan for AIDS Relief; STI: sexually transmitted infection
UNAIDS: Joint United Nations Programme on HIV/AIDS
USAID: United States Agency for International Development
VMMC: voluntary medical male circumcision
WHO: World Health Organization
