# Supplementary material for: Voluntary Medical Male Circumcision: Modeling the Impact and Cost of Expanding Male Circumcision for HIV Prevention in Eastern and Southern Africa
Source: PLoS Med. 2011 Nov 29;8(11):e1001132. doi: 10.1371/journal.pmed.1001132 (PMC3226464; doi:10.1371/journal.pmed.1001132)
Supplement: Table S1 — Model details and equations. (DOCX) [file pmed.1001132.s001.docx]

Table S1 - Model details and equations

The calculations are implemented in a typical model of infectious diseases modified to incorporate the effects of male circumcision and behavior change. The population is disaggregated by sex and age group (15-24 and 25-49). For each population group we describe a susceptible population, S_a,s,t_, that is increased by people aging into the population group (15 years olds entering the younger age group and 25 years olds leaving the younger age group and entering the older age group) and decreased by non-AIDS deaths and new infections (equation [1] below). There is also an infected population, I_a,s,t_, that is increased by new infections and decreased by non-AIDS deaths, AIDS deaths and aging out (equation [2] below).

HIV incidence (the proportion of the susceptible population becoming infected each year) is determined as the product of HIV prevalence in the population and the force of infection. The force of infection is determined by the base rate of infection (which is a fitting parameter and may vary by age and sex), changes in behavior and changes due to male circumcision. The effect of male circumcision on the force of infection is simply the increase in the prevalence of male circumcision multiplied by the reduction in susceptibility due to circumcision (assumed to be 60% in our analysis). Changes in behavior are assumed to occur naturally as the epidemic progresses due to two key influences. [1] Those with the riskiest behaviors will become infected first and die sooner than the rest of the population. Thus, even if no individual changes behavior the average behavior will become safer because of the death of those with the riskiest behaviors. [2] As AIDS deaths accumulate there can be a powerful effect on individual behavior, as those who know someone who has died from AIDS are motivated to adopt safer behaviors. Thus, the force of infection can drop over time as the cumulative number of AIDS deaths increases. The amount of the effect is determined by a fitting parameter that produces the best fit to the historical prevalence trend.

In the base case new infections increase over time in response to the initial force of infection. This causes HIV prevalence to rise rapidly in the early years. After some time the force of infection declines somewhat as AIDS deaths accumulate, and prevalence stabilizes. Implementation of a male circumcision program can cause a further reduction in the force of infection (depending on the increase in circumcision coverage) and lead to a decline in new infections and HIV prevalence.

The effects of other prevention programs can be incorporated through exogenous reductions in the force of infection. The effects of targeting male circumcision programs can be examined through increasing coverage in only one or both age groups for males or through neonatal circumcision which affects the circumcision status of 15 year olds entering the adult population 15 years later.

Descriptions of the variables and parameters used in the model and the model equations are provided below.

**Population group constants**

a = age group: 15-24 or 25-49

s = sex: male or female

s' = opposite sex

P = Population target group (adult males, 15-24 year old males, Newborns, etc.)

**Time constants**

t = time

ts = length of time step (years)

**Epidemiological constants**

TransRed[P] = Reduction in the annual probability of infection due to male circumcision in population group P

**Cost constants**

AdultUserFee = Fee paid per adult circumcision

CostPerAdultMC = Cost per circumcision for adult males

CostPerNewBornMC = Cost per circumcision for Newborns

NewBornUserFee = Fee paid per Newborn circumcision

**Model variables**

A[a,s,t] = AIDS deaths in age group a, sex s at time t

AIDS[a,s,t] = AIDS deaths to age group a, sex s at time t

B[s,t] = Births of sex s at time t

CBR[s,t] = Crude birth rate for sex s at time t expressed as births per 1000 adults

CumAIDS[a,s,t] = cumulative AIDS deaths to age group a and sex s at time t

e[a,s,t] = risk due to individual behavior as a proportion of the initial risk

E[s,t] = 15 year olds entering the adult population of sex s at time t

I[a,s,t] = Infection population in age group a, sex s, at time t

i[t] = pulse of new infections in the first year of the epidemic (as proportion of susceptible population)

MCr[a,s,t] = base force of infection as modified by changes in the prevalence of male circumcision

N[a,s,t] = Total population in age group a, sex s, at time t

NewI[a,s,t] = new HIV infections in age group a, sex s and time t

NewI'[a,s,t] = New HIV infections in age group a, sex s and time t without any change in MC prevalence

prev[a,s,t] = HIV prevalence in age group a, sex s and time t

psc[a,a1,s] = the proportion of all sexual contacts among adults that are between age group a and age group a1 of the opposite sex

r[a,s,t] = force of infection for age group a, sex s and time t

S[a,s,t] = Susceptible population in age group a, sex s, at time t

u[a] = Non-AIDS mortality rate in age group a

y[a,s,t] = Rate of new infections for age group a, sex s at time t

Model fit parameters

alpha[a,s] = parameter describing the rate of decline in the average risk of the susceptible population as prevalence increases. The decline occurs because those with the highest risk become infected first.

e'[a,s] = behavior change parameter that describes the decline in the average risk of the susceptible population due to individual behavior change

r'[a,s] = the force of infection at the start of the epidemic in age group a and sex s

**Equations**

[1] S[15-24,s,t] = S[15-24,s,t-1] + E[s,t] - (u[a] + y[15-24,s,t] + 1/10) x S[15-24,s,t-1]) x ts

The susceptible population aged 15-24 at time t is equal to the population at time t-1 plus new 15 year olds entering theadult population minus deaths, new infections and those becoming age 25, estimated as 1/10 of the population.

[2] I[15-24,s,t] = I[15-24,s,t-1] + (y[15-24,s,t] + i[t]) x S[15-24,s,t-1] - (u[a] + 1/10) x I[15-24,s,t-1] x ts - A[15-24,s,t]

The infected population aged 15-24 at time t is equal to the population at time t-1 plus new infections minusnon-AIDS deaths and those becoming age 25, estimated as 1/10 of the population, minus AIDS deaths.

[3] N[15-24,s,t] = S[15-24,s,t] + I[15-24,s,t]

The total population aged 15-24 is equal to the susceptible population plus the infected population

[4] E[s,t] = B[s,t-15] * S15

The number of 15 years olds in year t is equal to the number of births 15 years ago multiplied by the proportion surviving to age 15

[5] B[s,t] = (N[15-24,s,t] + N[25-29,s,t]) / 1000 * CBR[s,t] x ts

[6] y[15-24,s,t] = r[a,s,t] * (prev[15-24,s',t-1] x psc[15-24,15-24,s'] + prev[25-49,s',t-1] x psc[15-24,25-49,s'] / (psc(15-24,15-24,s'] + psc[15-24-25-49,s'])

The rate of new infections is equal to the force of infection times the weighted average of prevalence in the opposite sexwhere the prevalence is weighted by the proportion of contacts with each age group.

[7] r[a,s,t] = MCr[a,s,t] x exp(-alpha[a] x prev[a,s,t-1]) x e[a,s,t]

The force of infection is equal to the base force of infection, MCr, multiplied by a factor that expresses the reduction in the average risk of the population as prevalence increases, expressed as an exponential function of prevalence and a constant alpha determined by the model fit, multiplied by the behavior change variable.

[8] MCr[a,s,t] = r'[a,s] * ( (1-Mcprev[a,s,t]) + ∑(P) (NewMC%[a,s,t,P] * TransRed[P])

The base force of infection is equal to the initial force of infection determined by the model fit to historical data modified byany changes in the prevalence of male circumcision, calculated as the sum of the change in prevalence of MC in each targetpopulation group (P) multiplied by the reduction in transmission among circumcised men in that population group.

[9] e[a,s,t] = exp( -e'[a,s] x CumAIDS[a,s,t])

The reduction in the force of infection due to behavior change is described as an exponential function of cumulative AIDS deaths.

[10] NewI[a,s,t] = (y[a,s,t] + i[t]) * S[a,s,t-1]

New infections are equal to the rate of new infections plus any pulse of infections to start the epidemic multiplied by the susceptible population.

[11] AIDS[a,s,t] = ∑(T) NewI[T] x (CAM[t-T] - CAM[t-T-1])

AIDS deaths are calculated as the sum over all years from the start of the epidemic to time t of the product of new infections t-T years agoand the difference in the cumulative proportion surviving T and T-1 years after infection.

[12] MCcost[t] = NewNewBornMC[t] x CostPerNewBornMC - NewNornUserFee) + (NewMC[t] - NewNewBornMC[t]) * (CostPerAdultMC - AdultUserFee)

The net cost of male circumcisions in year t is the number of circumcisions among newborns multiplied by the net cost ofNewborn circumcision (full cost minus any fees) plus the number of adult circumcisions multiplied by the net costof adult circumcisions.

[13] CostPerInfectionAverted = ∑(t) (MCcost[t] x InfectionsAverted[t])

The cost per infection averted is the sum of costs divided by the sum of infections averted

[14] InfectionsAverted[t] = NewI'[a,s,t] - NewI[a,s,t]

Infections averted in year t is the number of new infections in year t without any increase in MC minus the new infections with the increase in MC
